# pH-Selective Reactions to Selectively Reduce Cancer Cell Proliferation: Effect of CaS Nanostructures in Human Skin Melanoma and Benign Fibroblasts

**DOI:** 10.3390/biochem3010002

**Published:** 2023-01-18

**Authors:** Olga M. Rodríguez Martínez, Michelle A. Narváez Ramos, Angeliz A. Soto Acevedo, Carolina C. Colón Colón, Darlene Malavé Ramos, Coral Castro Rivera, Miguel E. Castro Rosario

**Affiliations:** 1 Department of Biology, School of Arts and Sciences, University of Puerto Rico at Mayaguez, Mayaguez, PR 00682, USA; 2 Department of Chemistry, School of Arts and Sciences, University of Puerto Rico at Mayaguez, Mayaguez, PR 00682, USA

**Keywords:** melanoma, calcium sulfide nanostructures, extracellular pH

## Abstract

An acidic extracellular pH value (pH_e_) is characteristic of many cancers, in contrast to the physiologic pH_e_ found in most benign cells. This difference in pH offers a unique opportunity to design and engineer chemicals that can be employed for pH-selective reactions in the extracellular fluid of cancer cells. The viability of human skin melanoma and corresponding fibroblasts exposed to CaS dispersions is reported. The viability of melanoma cells decreases with CaS dispersion concentration and reaches 57% at 3%, a value easily distinguishable from melanoma control experiments. In contrast, the viability of benign fibroblasts remains nearly constant within experimental error over the range of dispersion concentrations studied. The CaS dispersions facilitate vinculin delocalization in the cytoplasmic fluid, a result consistent with improved focal adhesion kinase (FAK) regulation in melanoma cells. Thermodynamic considerations are consistent with the formation of $H_{2}S$ from CaS in the presence of protons. The thermodynamic prediction is verified in independent experiments with solid CaS and acidic aqueous solutions. The amount of $H_{2}S$ formed decreases with pH. An activation energy for the process of (30 ± 10) kJ/mol in the temperature range of 280 to 330 K is estimated from initial rate measurements as a function of temperature. The total Gibbs energy minimization approach was employed to establish the distribution of sulfides—including $H_{2}S$ in the gas and aqueous phases—from the dissociation of CaS as a function of pH to mimic physiologically relevant pH values. Theoretical calculations suggest that partially protonated CaS in solution can be stable until the sulfur atom bonds to two hydrogen atoms, resulting in the formation of Ca^2+^ and $H_{2}S$, which can be solvated and/or released to the gas phase. Our results are consistent with a model in which CaS is dissociated in the extracellular fluid of melanoma cells selectively. The results are discussed in the context of the potential biomedical applications of CaS dispersions in cancer therapies.

## 1. Introduction

Maintenance of the intracellular concentration of protons (H^+^) is central to cell survival. Protons are generated by processes inside the cell, notably glycolysis [1–5]. Buffers in the intracellular fluid and transport proteins in the membrane handle fluctuations in the pH with various mechanisms, resulting in a basic intracellular pH (pH_i_ ). In the case of benign cells, the extracellular pH (pH_e_) is also basic, owing to various buffer and proton exchange mechanisms. On the other hand, in cancer cells, their metabolic rate is significantly higher than that in benign cells, resulting in a higher rate of proton formation in the intracellular fluid [6,7]. Cancer cells manage to maintain the basic pH_i_ required to survive by increasing the number and functions of transport mechanisms from the intracellular to extracellular fluid. These mechanisms facilitate cancer cells to maintain a basic pH_i_ at the expense of an acidic pH_e_. Indeed, an acidic pH_e_ microenvironment is usually considered one of the hallmarks of cancer cells [6].

Recent computational work by Persi and collaborators has pointed out that a basic pH_i_ is required to facilitate cancer cell proliferation, while achieving the opposite, an acidic pH_i_ value, results in a reduction in cancer cell proliferation [8]. As discussed in earlier work by Koltai, targeting tumor pH values may allow control of cancer cell proliferation. Swietach and coworkers have developed a mathematical framework and performed simulations on pH_i_ and pH_e_ of cancer cells and tumors [7,9]. Their results point out the importance of various buffers and transport mechanisms—including lactic acid and CO_2_/HCO_3_^−^/H^+^—to regulate pH. The above authors have pointed out the importance of understanding the mechanisms of acid handling to identify a vulnerability that could be extended to new therapeutics. The results discussed above have opened up a new possibility to develop strategies that could reduce the number of mechanisms employed by cancer cells to maintain the delicate basic pH_i_.

The difference in pH_e_ is also a potential alternative with which to develop new therapeutics that can undergo reactions in cancer cells selectively [10]. Reactions that can selectively produce apoptotic agents are expected to reduce cancer cell proliferation with little effect on normal cells. Calcium sulfide (CaS) may be an excellent candidate to accomplish this task.

In this work, we report the effect of CaS dispersions on the viability of human skin melanoma and benign cells. In a previous work from our group, the dissociation of calcium sulfide in acidic environments was explored experimentally as a potential strategy to selectively reduce the proliferation of human breast cancer cells in vitro [11,12]. The standard Gibbs free energies of formation of the relevant chemicals are summarized in Table 1. The standard Gibbs free energy change (ΔG^0^) for the reaction:

(Reaction 1) $${CaS}_{\left(aq\right)}\to {Ca}^{2+}{\;}_{\left(aq\right)}+S^{-2}{\;}_{\left(aq\right)}$$

is +109 kJ/mol with an equilibrium constant of the order of 10^−19^. In the presence of protons, the reactions:

(Reaction 2a) $${CaS}_{\left(aq\right)}+2H^{+}{\;}_{\left(aq\right)}\to {Ca}^{2+}{\;}_{\left(aq\right)}+H_{2}S_{\left(aq\right)}$$

(Reaction 2b) $${CaS}_{\left(aq\right)}+2H^{+}{\;}_{\left(aq\right)}\to {Ca}^{2+}{\;}_{\left(aq\right)}+H_{2}S_{\left(gas\right)}↑$$

have standard reaction Gibbs free energy changes of −108 and −113 kJ/mol, respectively. The negative sign of the standard Gibbs free energy changes indicates that Reactions (2a) and (2b) are highly spontaneous. Thus, in the acidic extracellular environment that characterizes cancer cells, it is expected that Reaction (2) will result in the formation of ${Ca}^{2+}{\;}_{\left(aq\right)}$ and $H_{2}{\;S}_{\left(aq\right)}$, while the more basic extracellular environment found in benign cells limits the extent to which Reaction (2) takes place. $H_{2}S$ has been proposed to play important roles in several physiological processes and small amounts can cause programmed death in several cellular systems. ${Ca}^{2+}$ ions in the extracellular fluid in concentrations higher than 500 mM are also known to cause apoptosis.

This work reports the effect of CaS dispersions in the proliferation of human melanoma and benign epidermal cells in vitro. The skin has a thin acidic mantle that consists of a hydrolipidic film and dead skin cells. The living cells that constitute the skin epidermis are located below this protective layer and have a basic pH of about 7.4. Melanocytes—the cells that give rise to skin color—are in the epidermis. Skin melanoma results from anormal growth of melanocyte cells. The extracellular pH of melanoma cells in malignant tumors is lower than 7, in contrast to the basic pH value found in benign cells [13]. The work presented here adds nicely to our previous works on human breast adenocarcinoma and reports quantitative measurements of CaS dissociation in acidic media. The results of the first 24 h following a single dose indicates viability as low as 57% of skin melanoma cells treated with a 3% dispersion concentration. This is a notable contrast with the corresponding normal cells, which have a viability between 100 and 87%. It is proposed that the acidic microenvironment in the extracellular fluid of melanoma cells facilitates the formation of free ${Ca}^{2+}$ ions and sulfides—including $H_{2}S$ and HS^−^ ions.

## 2. Methodology and Approach

### Cell viability measurements:

Human skin melanoma adherent cell cultures were prepared from the ATCC Hs 895.T CLR-7637 line. Human skin normal fibroblasts were prepared from the ATCC CCD 1090 sk CRL-2106 cell line. Cell culture samples were prepared in 96-well microtiter plate (Costar) and placed in an incubator for the desired period of time. The media used for melanoma and benign skin cells are different. Both of these media have a pH indicator—phenol red. The indicator changes to the orange of slightly acidic media almost immediately after the cells are added to the media for passages or when the media is added to the melanoma cell culture. This is not observed with the benign cells and the corresponding media; they keep the reddish color typical of basic pH values. The cell cultures were stained with Hoechst/PI solution after 24 h of incubation and imaged in a digital fluorescence and confocal microscope with 4× magnification and analyzed with the microscope’s Cell Reporter Xpress Pico Image System (Molecular Devices, San Jose, CA, USA) for cell viability measurements. The number of cells alive was determined from the difference between the total number of cells and number of dead cells. Quantitative analyses were obtained with GraphPad software (Boston, MA, USA). The reported viability references the ratio of the number of live cells in the sample to the corresponding number in the control experiment. Error bars represent the standard deviation from the average obtained from the analysis of three to five measurements at the indicated dispersion concentration.

### Vinculin expression:

Cells were immunostained with the Actin Cytoskeleton and Focal Adhesion Staining kit (Millipore Sigma FAK100, Sigma Aldrich, Saint Louis, MO, USA) using the Fluorescein (FITC) AffiniPure Goat Anti-Mouse IgG (H+L) 2 mg—115–095-003 (Jackson Immuno Research, West Grove, PA, USA) with the following modifications: the antibodies were diluted in 1% BSA, 0.1% Tween^®^−20 and PBS1X and primary antibodies were incubated overnight instead of for 4 h. An optimum working concentration of 1:250 was determined based on personal criteria of adequate cell image with an exposure time of 400 milliseconds. Reported three-dimensional (3D) cell image measurements were performed from a stack of 60 images with an Olympus confocal microscope using a 60× magnification lens, with an excitation wavelength of 492 nm and an emission wavelength of 518 nm for the secondary antibody (FITC).

### CaS Dispersion preparation:

CaS dispersions were adapted and modified from previous studies [14]. Briefly, a trace amount of laboratory-grade calcium acetate (Ca(CH_3_CO_2_^−^)_2_), purchased from Fisher Scientific, was dissolved with 5 mL of dimethyl sulfide (DMSO) and the resulting dispersion was placed in a commercial microwave oven and warmed in intervals of 5 s until a total time of 30 s was accumulated. The resulting dispersion was found to be slightly yellow.

### Experimental $H_{2}S$ formation and related kinetics from CaS:

The amount of $H_{2}S$ formed from the reaction of solid CaS with acidic aqueous solutions was determined from the reaction of CaS(s) with acidic solution using a BW Technologies Honeywell sensor. A Mettler Toledo AT20 analytical balance was used to determine the amount of CaS(s). The CaS was placed in a 5 mL cylindrical glass vial used as a reactor. The reactor was closed with a plastic cap that allows for a syringe to inject 1 mL of an HCl acidic solution of the desired pH. HCl solutions were prepared by diluting a 0.90 M HCl solution with enough water until the desired pH value was obtained. N_2_ was used to stir the reaction mixture and a carrier gas from the reactor to the $H_{2}S$ detector. One end of a $\frac{1}{4}$ inch Teflon tube was connected to an N_2_ gas regulator and the other end to a needle inserted into the reactor. The N_2_ gas was allowed into the reactor at a slow rate (about 0.25 L/min). The exit gas line consisted of a separate $\frac{1}{4}$ inch Teflon tubing coupled to the calibrated $H_{2}S$ sensor by one end and to the reactor on the other end. The ppm of $H_{2}S$ displayed in the detector was video-recorded in real time. The reactor assembly was immersed to an appropriate level in a temperature-controlled water bath. The temperature was measured with a standard thermometer placed in the bath. The ppm of $H_{2}S$ as a function of time was obtained from analysis of the video-recorded information on a personal computer using the slow-motion option in photo applications that are part of the Dell computer system package and translated to an Excel or Igor worksheet for further analysis. Experimental determination of the total amount of $H_{2}S$ formed was determined in a separate experiment. The $H_{2}S$ formed was allowed to accumulate in the reactor for 15 min. A Hamilton gas-tight syringe was used to collect the gas formed. The collected gas was then injected into a homemade gas reactor directly coupled to the detector and reported as the maximum amount of $H_{2}S$ formed. The solubility of $H_{2}S$ in water may be significant at the temperatures studied here. Thus, the parts per million (ppm) of $H_{2}S$ reported here represent a lower limit of the total gaseous $H_{2}S$ formed.

### Gibbs energy minimization:

Total Gibbs free energy calculations were performed on a personal computer on the Python platform. Attempts were made to run the calculations with the use of Excel Solver and/or MATLAB platforms with little success. The Python platform provided reliable and consistent results. The equilibrium amounts and pressures were validated by comparison with the equilibrium constants determined from standard Gibbs free energies. The results predict equilibrium constants that are between 5% and 20% of the standard equilibrium constants. The larger deviations were observed when dealing with smaller amounts and concentrations of chemical species.

**Theoretical calculations** related to the chemistry of the CaS nanoclusters were performed with the Gaussian 16W package using the GaussView 6.0/16 environment. The structures were optimized at the DFT /B3LYP/DFT level of theory using the DGZVP basis set. Only water-solvated optimized structures with positive vibrational frequencies are reported in this work.

## 3. Results

### Cell viability studies:

**Figure 1.**
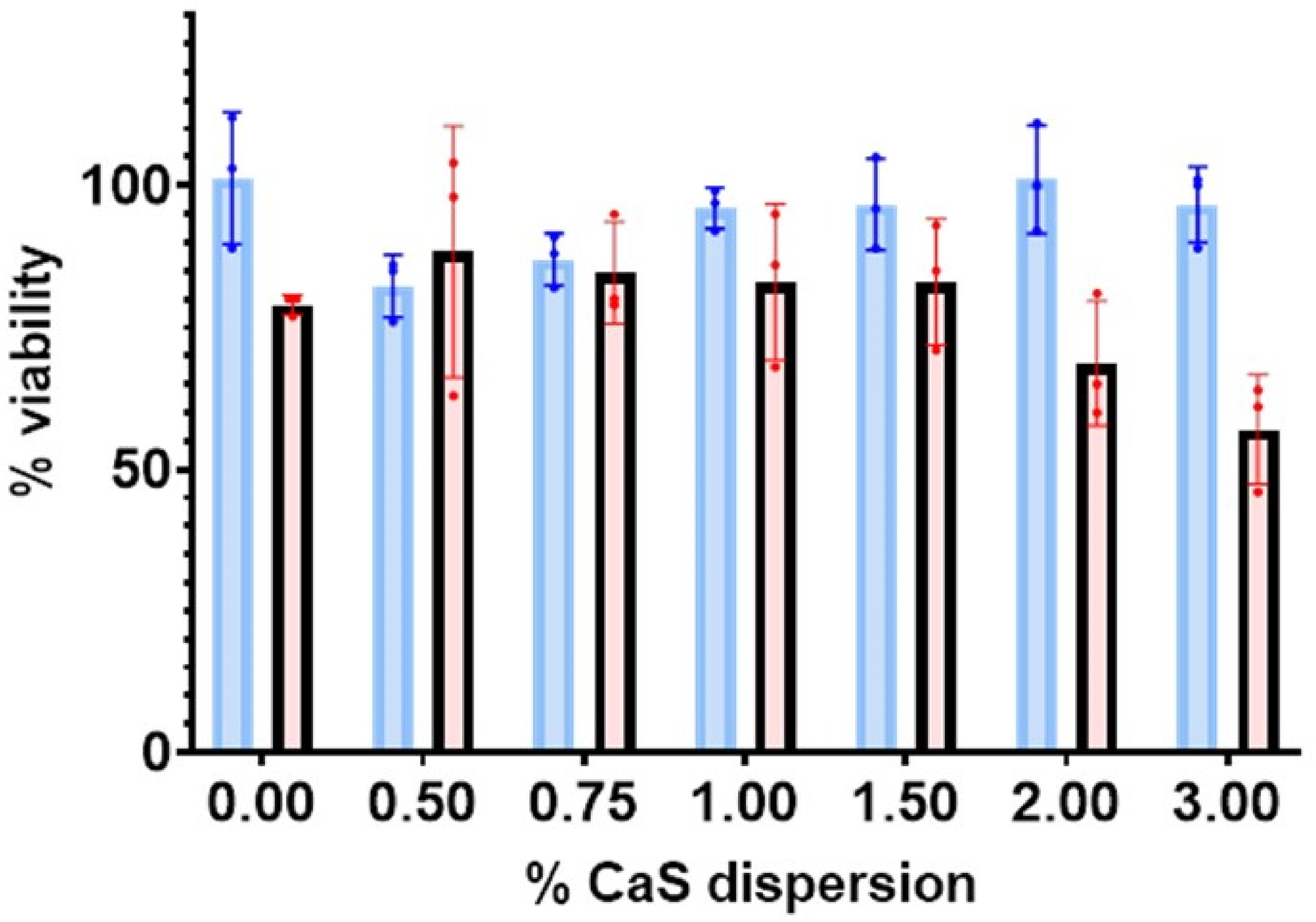
The percentage of live malignant (red) and benign fibroblasts (blue) as a function of the percentage (%) of CaS dispersion used.

The results of the 24 h viability measurements are summarized in Figure 1. The viability of benign fibroblastic remains within 100% of the experimental error for the CaS dispersions employed. The viability is nearly constant within the experimental error for all dispersions employed in the measurements summarized in Figure 1 for benign fibroblasts. The average viability of melanoma cells, on the other hand, decreases with increasing CaS dispersion concentration and decreases to values as low as 57% for dispersion concentrations of 3%. The uncertainty in the viability obtained for initial CaS dispersion concentrations around 3% is well-separated from the 87% viability values reported for benign fibroblasts at the same concentrations, as well as the corresponding melanoma control. We conclude that the 3% CaS dispersions reduce the viability of melanoma cells with little effect on the viability of corresponding benign cells. The results are consistent with our earlier observations on human lung and breast cancer cell lines, where the CaS nanostructures reduce malignant cell proliferation with little or no effect on corresponding benign cells [15–18].

**Figure 2.**
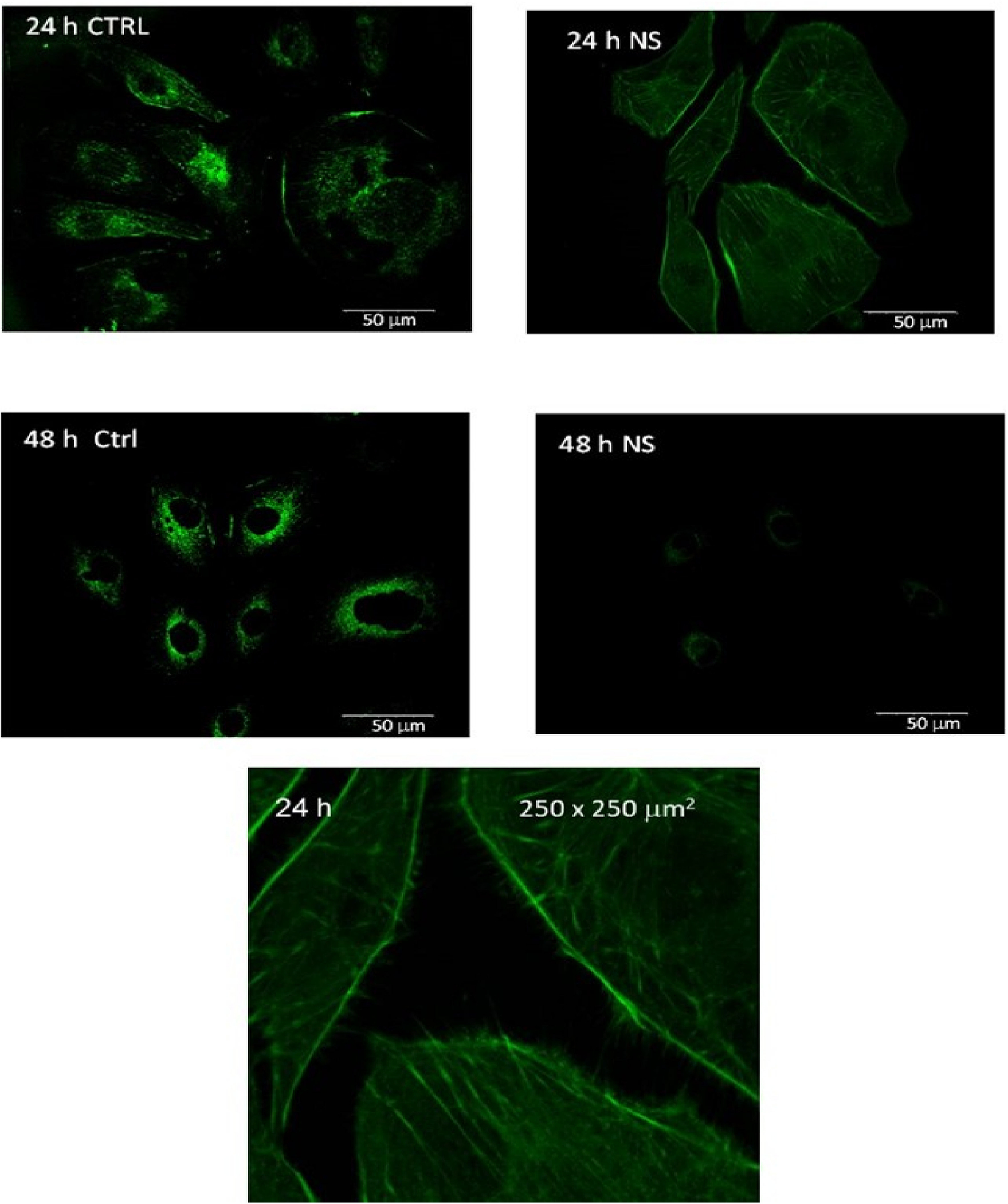
Vinculin expression in the melanoma control (CTRL) and 24 and 48 h after CaS dispersion treatment. The objective magnification is 60x. The image at the bottom represents a 250 × 250 μm^2^ region (magnified by a factor of about 4) of the 24 h measurement of the treated cells.

Images of labeled vinculin in control melanoma cells and 24 h- and 48 h-post-treatment melanoma cells are summarized in Figure 2. The spatial distribution of vinculin expression is found to be markedly different in the treated melanoma cells compared to the malignant control cells. The dispersion is found to deactivate and delocalize vinculin in the first 24 h post-treatment of the melanoma cells. Vinculin is found to localize only around the nuclei 48 h post-treatment with a significantly lower expression intensity than that in the corresponding melanoma control.

### pH-dependent measurements of $H_{2}S$ from CaS:

**Figure 3.**
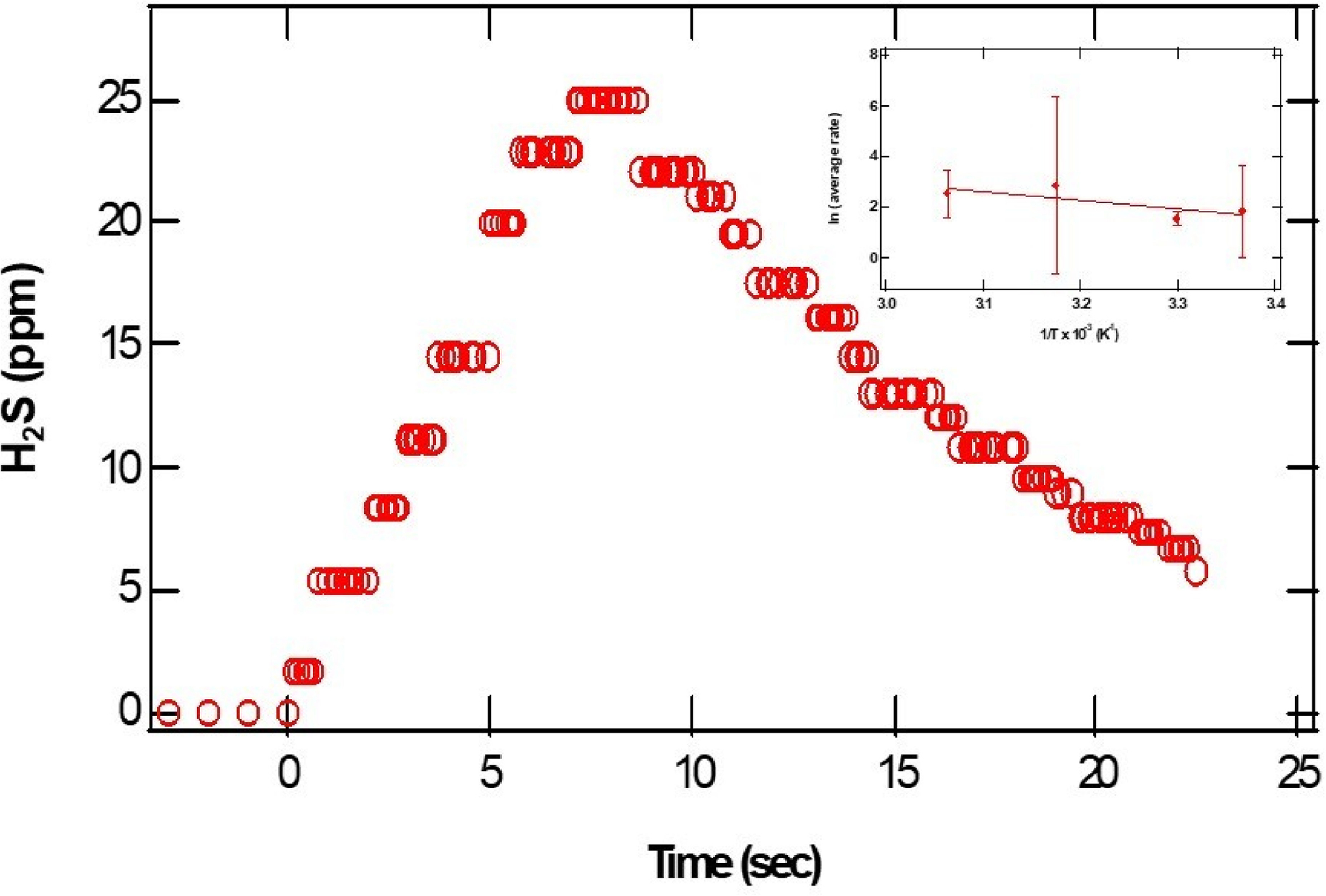
The open circles represent the parts per million of $H_{2}S$ detected as a function of time. The pH of the acidic solution employed was 0.70. The initial amount of CaS(s) is 0.01 mg. The closed circles in the insert on the upper right-hand side represent the In(r) as a function of 1/T.

The dense dots in Figure 3 represent the parts per million of $H_{2}S$ gas detected at the exit of the reactor as a function of time. From the initial rise in the ppm of $H_{2}S$, we estimated relative initial reaction rates. The insert represents a plot of the natural logarithm of initial reaction rates as a function of the reciprocal temperature. The error bars represent the standard deviations of at least three measurements at about the same temperature. Assuming a rate law of the form:

(1) $$r={Ae}^{-Ea/RT}{\left[H^{+}\right]}^{a}[CaS{]}^{b}$$

where A represents the pre-exponential factor, $E_{a}$ represents the activation energy for the process, and R and T represent the universal gas constant and temperature in K, the values of a and b correspond to the reaction orders of $H^{+}$ and CaS, respectively. From the slope of the plot displayed in the insert of Figure 3, we estimate an activation energy of (30 ± 10) kJ/mol.

**Figure 4.**
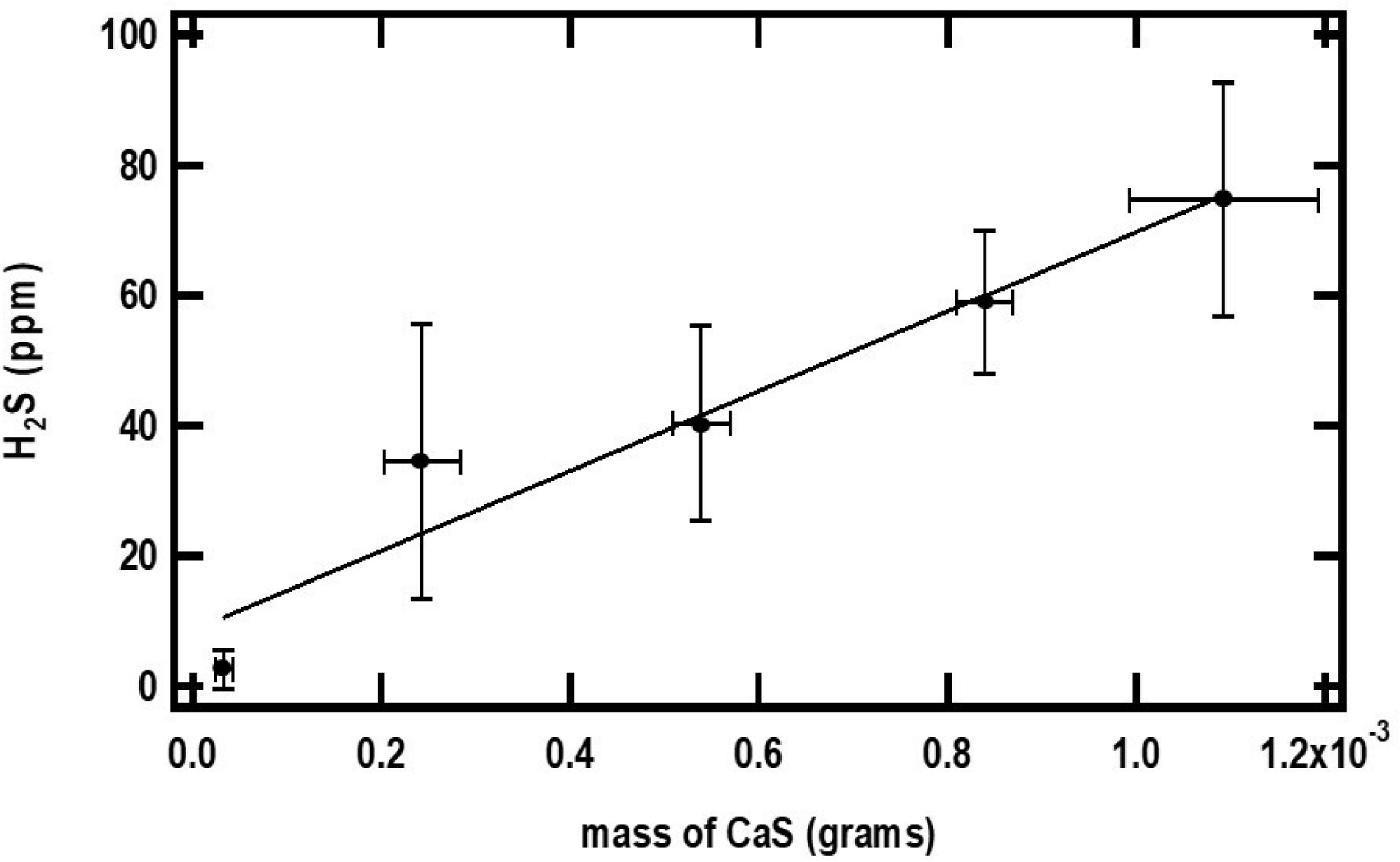
The small black dots in the figure represent the maximum amount of $H_{2}S$ detected (ppm) as a function of the initial amount of CaS(s). The error bars represent the standard deviation of at least three measurements with similar amounts of CaS(s). The pH of the acidic solution employed is 0.70. See text for details.

**Figure 5.**
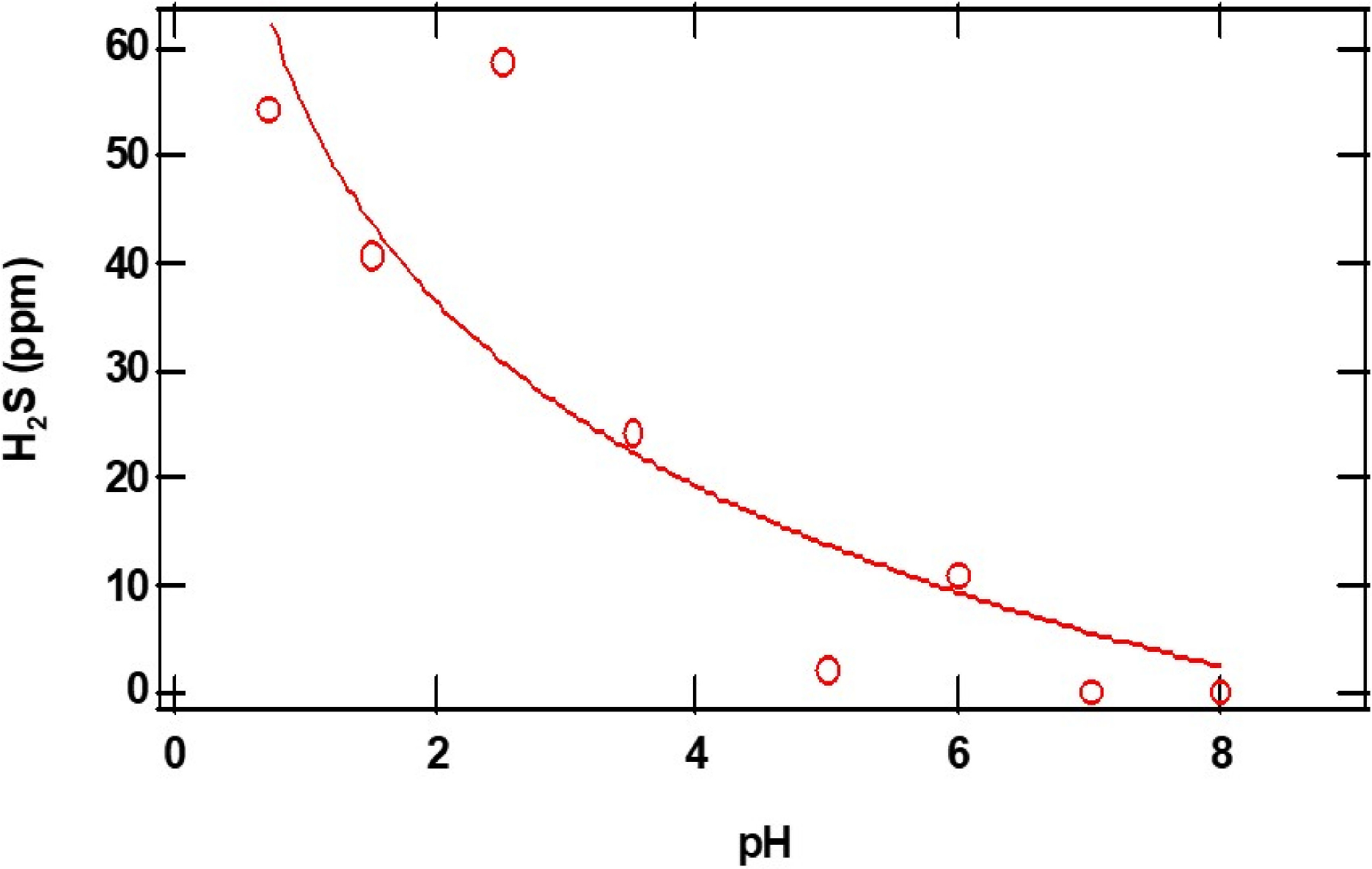
The open red circles in the figure represent the maximum amount of $H_{2}S$ detected (ppm) as a function of initial pH. The initial amount of CaS(s) employed is 0.01 mg. See text for details.

The closed and open circles in Figures 4 and 5 represent the ppm of $H_{2}S$ formed as a function of the initial amounts of CaS and pH, respectively. The amount of $H_{2}S$ formed increases monotonically with the initial amount of CaS. The ppm of $H_{2}S$ detected, on the other hand, decreases with pH. These observations are consistent with Reactions (1) and (2) for CaS in proton-poor and proton-rich environments, respectively.

### Determination of sulfide distribution:

We studied the distribution of sulfides as a function of pH to learn about the chemistry of sulfides in the extracellular environment of cancer cells and benign cells. Sulfides are known to establish multiple equilibria in aqueous solution according to the set of Reactions (3a) to (3c) below:

(Reaction 3a) $$H_{2}S_{\left(aq\right)}⇄H_{2}S_{\left(gas\right)}$$

(Reaction 3b) $$H_{2}S_{\left(aq\right)}+H_{2}O_{\left(liq\right)}⇄{HS}^{-}{\;}_{\left(aq\right)}+H_{3}O^{+}{\;}_{\left(aq\right)}$$

(Reaction 3c) $${HS}^{-}{\;}_{\left(aq\right)}+H_{2}O_{\left(liq\right)}⇄S^{-2}{\;}_{\left(aq\right)}+H_{3}O^{+}{\;}_{\left(aq\right)}$$

(Reaction 3d) $$H_{3}O^{+}{\;}_{\left(aq\right)}+{OH}^{-}{\;}_{\left(aq\right)}⇄2H_{2}O_{\left(liq\right)}$$

The use of equilibrium constants to obtain equilibrium concentrations is of little use due to the complexity added by the mixed appearance of chemicals as reactants and products in the multiple-equilibria set of reactions presented above. A computer program was developed in the Python environment to establish the distribution of sulfides using the minimization of the total Gibbs energy of the system in solution. The strategy is summarized in Figure 4 [19–22]. The program minimizes the total Gibbs free energy in solution considering all chemical species, including $H_{3}O^{+}$ and ${OH}^{-}$ and the water solvent, as well as gaseous $H_{2}S$. Briefly, the Gibbs free energy of a the *j*th chemical species in the mixture is given by:

(2) $$G_{mj}={G_{mj}}^{{}^{\circ}}+RT\;ln\left(P_{j}/P^{{}^{\circ}}\right)$$

for gases, and

(3) $$G_{mj}={G_{mj}}^{*}+RT\;lnx_{j}$$

for species in solution. In Equation (2), ${G_{mj}}^{{}^{\circ}}$ and $P_{j}$ represent the standard free energy and the partial pressure of the j^th^ gas, respectively. ${G_{m}}^{*}$ and $x_{j}$ in Equation (3) represent the standard Gibbs free energy and the mole fraction of the j^th^ species in solution, respectively. In a multicomponent system containing j species, the total Gibbs energy reaches a minimum when the system reaches equilibrium:

(4) $${dG}_{\text{total}\;}=\sum_{j}G_{mj}{dn}_{j}=0$$

This relation has been employed in the past to establish equilibrium concentrations in several simple systems, notably in the production of hydrogen from methane for fuel cells, and in the synthesis of ammonia [20,21]. The Gibbs free energy minimization approach establishes equilibrium concentrations from the constituents’ elements that are in a system under a mass balance restriction. The mass balance restriction requires that the difference between the number of atoms of the j^th^ element distributed among the different $a_{i}$ equilibrium products and the corresponding initial number of atoms ($b_{j}$) is zero:

(5) $$\sum_{i,j}\left(n_{j}a_{i,j}-b_{j}\right)=0$$

for the subscript j run over the atoms and the subscript i run over the chemical species. The $n_{j}$ represents the number of moles of j atoms in the $i^{\text{th}\;}$ chemical species with an overall number of moles $a_{ij}$, and the $b_{j}$ represents the initial number of $j^{\text{th}\;}$ atoms in the system. In addition to the mass balance constraint, we add the requirement that the equilibrium concentrations obtained match the known equilibrium constants obtained from the traditional relation:

$$ln(K)=-\Delta {G^{{}^{\circ}}}_{rxn}/RT\;\text{for}\;\text{any}\;\text{biochemical}\;\text{reaction}\;\text{or}\;\text{process}$$

where the $\Delta {G^{{}^{\circ}}}_{rxn}$ is obtained from the accepted Gibbs free energy of formation of participating species. To our knowledge, none of the software available fulfills these two constraints simultaneously.

**Figure 6.**
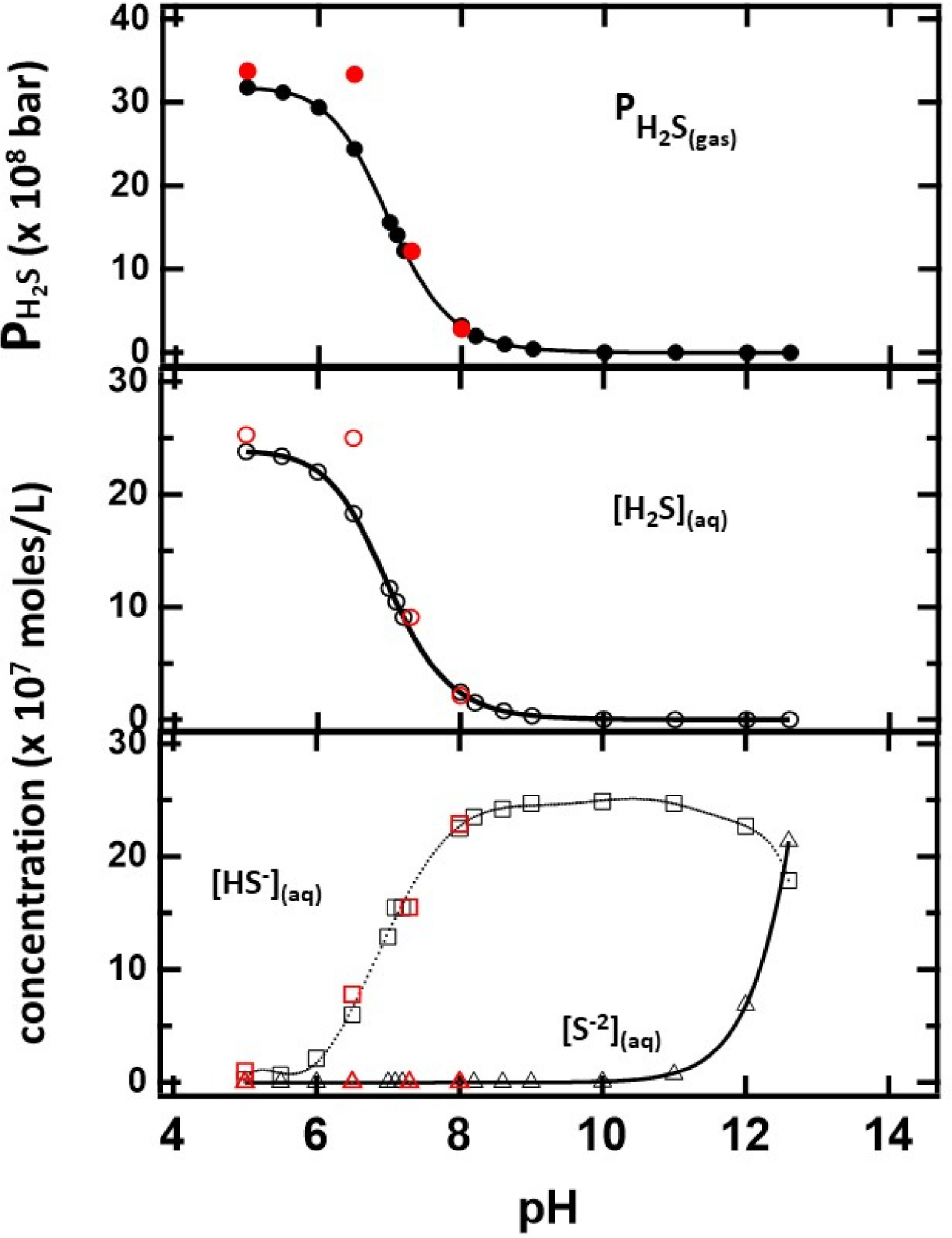
The distribution of sulfides in the gas and solution phases as a function of pH determined from minimization of the Gibbs free energy. The number of moles of sulfur is 2.5 × 10^−6^ moles for the indicated pH values. Values obtained from identical CaS amounts are indicated in red.

The results of the distribution of sulfides as a function of pH are summarized in Figure 6. The machine learning component of the program predicts average equilibrium constants for $H_{2}S$ and HS^-−^ dissociation K_a1_ = (9.0 ± 0.5) × 10^−8^ mol/L and K_a2_ = (10 ± 2) × 10^−14^ mol/L, respectively, and K_w_ = (1.0 ± 0.2) × 10^−14^ mol^2^/L^2^ for water autoionization. These values are within the uncertainty of the calculation and in close agreement with accepted values. The solubility of ${H_{2}S}_{\left(\text{gas}\right)}$ in the solution is predicted to be 13.4 bar/mol/L, which is within 10% of the reported value. The concentration of $H_{2}S_{\left(aq\right)}$ and $H_{2}S_{\left(g\right)}$ pressure decreased with pH, consistent with the experimental results discussed earlier. The equilibrium concentrations of HS^-−^ and S^−2^, on the other hand, increase with the basicity of the solution. In passing, the results are slightly different from those estimated from the Henderson–Hasselbalch equation, which does not consider the gas phase equilibrium of the fully protonated sulfides.

The red circles on the figure represent the distribution when CaS is used as the sulfide precursor. The number of moles at each pH value is identical to those used when the initial sulfide source was $H_{2}S$ or HS^−^ or S^−2^. The thermodynamic calculation indicates that CaS still dissociates at basic pH values, but the amount of $H_{2}S$ in solution and gas phases is significantly lower than at any acidic pH.

### Theoretical Calculations:

Theoretical calculations were performed to establish fundamental chemistry of CaS in acidic media. It is plausible that protonation results in the formation of localized S–H bonds in the CaS nanoclusters. Thus, the stability of single- and double-protonated sulfur atoms in the CaS nanoclusters and how it varies with possible experimental conditions are relevant to this work. The relevant results are summarized in Table 2. We found that the energy of the CaS decreases with the addition of one and two protons. The Ca–S bond length also increases from 2.549 A in CaS to 2.7323 and 3.125 A in CaSH^+^ and CaSH_2_^2+^, respectively.

## 4. Discussion

There has been significant interest in the chemistry of $H_{2}S$ in living cells, including cancer cells. This interest emerges from new developments related to the metabolism of sulfides in the body as well as the potential effect of reducing the proliferation of cancer cells. New fluorescent probes have been explored to detect $H_{2}S$ in living cells [23–26]. The dependence of the amount of $H_{2}S$ formed as a function of pH is consistent with our hypothesis that CaS easily dissociates in acidic environments. These results indicate that the amount of $H_{2}S$ can be six times higher at a pH of 1 than at higher pH values. The activation energy for $H_{2}S$ formation is found to be significant and likely reflects the notion that multiple steps are involved in CaS protonation and dissociation.

In that regard, the Gibbs free energy minimization results are revealing. The [S^−2^] concentration is not relevant until pH values above 10 units are reached. Those pH values are not relevant to many biological processes. A linear regression was used to fit the calculated values between pH = 6.6 and 7.4. In this range, the results for HS^-−^ and $H_{2}S$ concentrations and pressure were found to be fit adequately with linear equation of the form:

(6) $$[i^{\text{th}\;}\text{species}]=b+m\;*\;(pH)$$

resulting in values of the intercept and slope reported in Table 3. In this physiologically relevant pH region between 7.2 and 7.5 pH units, where benign fibroblasts are found in humans, the ${{\text{HS}}^{-}}_{\left(\text{aq}\right)}$ is the sulfide with the larger concentration in solution. At the pKa of $H_{2}S$ (7.01), ${{\text{HS}}^{-}}_{\left(\text{aq}\right)}$ is still the sulfide with the largest concentration in solution. This result contrasts with the predictions of the Henderson–Hasselbalch equation due to the presence of gas-phase $H_{2}S$(g), which reduces the amount of $H_{2}S$(aq). Based on the data presented in Figure 6, it is estimated that the $H_{2}S_{\left(aq\right)}$ and ${{\text{HS}}^{--}}_{\left(\text{aq}\right)}$ concentrations are equal—within 0.01 moles/L—when the pH is 6.948. The additional equilibrium involving $H_{2}S_{\left(g\right)}$ cannot be ignored in the design of a formulation based on calcium sulfide (or any sulfide) to reduce growth and proliferation of cancer cells. The pressure estimate is based on a large available volume. The total empty volume available to generate $H_{2}S_{\left(gas\right)}$ in the cell culture is unknown. In addition, certain solutes are known to affect $H_{2}S$ solubility in water. Since the energies associated with interactions between $H_{2}S$ and different areas of human cells are not known, the values provided here should be taken as a lower pressure limit.

The calculations allow us to consider the energetics of several simple processes that may be relevant to the reactions of CaS in acidic media. Table 4 summarizes calculated energies associated with the reactions of CaS and possible intermediates formed in acidic media. The calculations reveal that protonation releases energy, in agreement with the thermodynamic considerations discussed earlier in this work. It is also of interest to discuss the dependence of the Ca–S bond distance as a function of protonation. Bond lengths are usually taken as a measure of bond strength: the larger the bond length, the weaker the bond [27–30]. The bond length in sulfides ranges from 1.6 to about 2.9 Å [30]. As summarized in Table 2, the calculated Ca–S bond lengths in CaS and CaSH^+^ are within this range of values. The bond length in the double-protonated sulfide CaSH_2_^2+^, on the other hand, is outside of the range reported for sulfides. It is too large to allow for us to consider it a chemical interaction and leads us to conclude that double protonation results in the formation of ${Ca}^{2+}$ and $H_{2}S$. However, the calculations also indicate that the interaction between ${Ca}^{2+}$ ions and sulfur is strong enough to result in an optimized bond distance of about 3.13 Å. We take the difference in energy for the reaction leading to the formation of CaSH_2_^2+^ and the reaction leading to the formation of ${Ca}^{2+}$ and $H_{2}S$ as a measure of the interaction between ${Ca}^{2+}$ and $H_{2}S$. This energy difference is about 0.00203 hartrees, or 0.196 kJ/mol. At the physiologically relevant temperature of about 37 °C, there is enough energy (RT = 2.58 kJ/mol) to overcome this small barrier and facilitate the release of $H_{2}S$ and ${Ca}^{2+}$ ion mobility.

**Figure 7.**
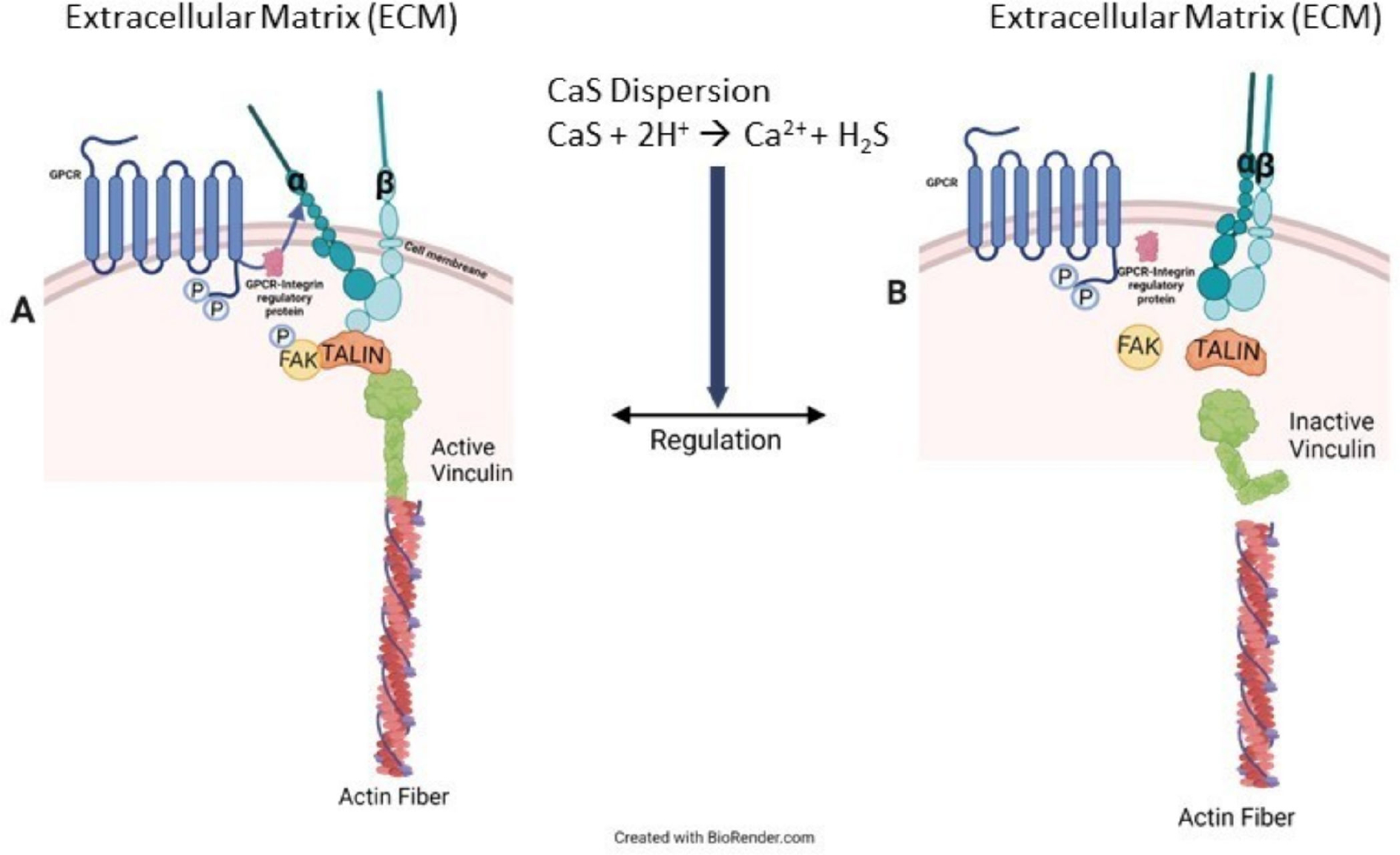
Cancer cells usually overexpress FAK, as illustrated in (**A**) on the left-hand side of the figure. This overexpression results in the formation of active integrin–FAK–talin–vinculin–actin focal adhesion points (FAP). CaS dispersion increases inactive vinculin in the cytoplasmic fluid, as in (**B**), which is attributed to the appropriate regulation of FAK and, consequently, the FAP formation mechanism. Proper FAK function also facilitates the activation of cell death mechanisms.

The overall results are consistent with our general hypothesis that CaS selectively dissociates in the acidic extracellular environment found in cancer cells—including lung, breast, and skin malignant tumors—but not in the fluid of corresponding benign cells [18]. The reader is referred to Figure 7 to facilitate the following discussion that represents our interpretation of a possible pathway to account for the observed results. The chemistry of the NS has a profound effect on the distribution of vinculin in the cell. Vinculin plays central roles in the formation of focal adhesion points [31]. Focal adhesion points form from complex interactions among transmembrane integrin and inner proteins that include talin, which recruits vinculin from the cytoplasmic fluid, focal adhesion kinases (FAK) and actin [32–36]. In benign cells, regulation of the focal adhesion point formation is largely due to activation and de-activation of FAK [37,38]. This regulation mechanism plays a central role in controlling cell death and proliferation [39,40]. In cancer cells, on the other hand, the mechanism is not properly regulated and FAK is overexpressed [39–41]. Control melanoma cells have significant focal points and vinculin around the nucleus, the latter likely resulting from interactions with talin localized in the cell nucleus [42,43]. In treated melanoma cells, we found vinculin to be delocalized over the entire cytoplasmic fluid, consistent with an increase in its inactive form, which is consistent with the activation of FAK regulation mechanisms [44]. We also observe an increase in the vinculin projection beyond the cell membrane, consistent with proper FAK functioning. Taken together, these results lead us to conclude that the chemistry of the NS formulation facilitates cell regulation associated with vinculin in the cytoplasmic fluid as well as the cell membrane. Details related to the specific effect of sulfides and calcium ions on the viability of melanoma cells exposed to CaS dispersion are beyond the scope of the work presented here. Such studies are important, since cell regulation in melanoma reduces cell viability, as observed in our experiments. Further speculation is unwarranted until experiments targeted at elucidating the role of sulfides and free calcium ions, as well as any other dispersion component—if any—in the cell death mechanism, are performed. Work in this direction is starting to take place in our laboratories and will be published in the near future.

## 5. Conclusions

In summary, we presented evidence for the selective reduction of melanoma cell proliferation as reflected in viability studies. Microscopy measurements are consistent with CaS dispersion facilitating vinculin delocalization. The selective formation of $H_{2}S$ and ${Ca}^{2+}$ ions from the reaction of CaS with protons is proposed to play a central role in the observed selectivity towards melanoma cells. Independent experiments and theoretical calculations are consistent with this interpretation. The amount of $H_{2}S$ released to the gas phase decreases with the pH of the aqueous solution. The activation energy for the process is estimated at (30 ± 10) kJ/mol in the temperature range of 280 to 330 K. $H_{2}S_{\left(gas\right)}$, $H_{2}S_{\left(aq\right)}$ and ${{\text{HS}}^{-}}_{\left(\text{aq}\right)}$ are the relevant species in solution in the physiologically relevant pH range of 6.6 to 7.5 according to the Gibbs energy minimization approach. Partially protonated CaS—only one hydrogen atom bonded to a sulfur atom—can be stable until two hydrogen atoms are bonded to the sulfur atom, resulting in the formation of ${Ca}^{2+}$ and $H_{2}S$, which can be solvated or released to the gas phase.

## Figures and Tables

**Table 1.** Thermodynamic values of the Gibbs free energy of formation of various calcium- and sulfur-containing species relevant to the discussion of the pH-sensitive reactions of CaS.

Species	DG_f_^0^ (kJ/mol)
Ca^2+^	−553.58
S^−2^	85.8
H^+^	0
CaS	−477.4
$H_{2}S$	−33.6

Thermodynamics in water: ${{\text{DG}}_{\text{rxn}}}^{0}(\;\text{kJ}/\text{mol})$. ${\text{CaS}}_{\left(\text{aq}\right)}\to {{\text{Ca}}^{2+}}_{\left(\text{aq}\right)}+{\text{S}}_{\left(\text{aq}\right)}^{-2}+9.62{\text{CaS}}_{\left(\text{aq}\right)}+2{{\text{H}}^{+}}_{\left(\text{aq}\right)}\to {{\text{Ca}}^{2+}}_{\left(\text{aq}\right)}+{\text{H}}_{2}{\text{S}}_{\left(\text{g}\right)}-109.74$.

**Table 2.** Energies associated with various sulfides in solution calculated at the DFT /B3LYP/DGZVP level of theory.

Chemical Species	Energy (Hartrees)	CaS Bond Length (A)	H-S Bond Length (A)
CaS	−1075.82635	2.54902	-
CaSH ^+^	−1076.311392	2.73303	1.35325
Ca-SH_2_^2+^	−1076.746989	3.12524	1.34873
HS^−^	−398.901543	-	1.35771
$H_{2}S$	−399.363436	-	1.34805
Ca^2+^	−677.38152	-	-
S^−2^	−398.377571	-	-

**Table 3.** Coefficients of a linear regression to the calculated [HS^−^] and [H_2_S] aqueous concentrations and the H_2_S gas partial pressure in the pH range of 6.6 to 7.5 units. The uncertainties in the values of b and m are indicated in the table. The units of b and m are moles/L in the case of the concentration of aqueous species and bar in the case of H_2_S gas.

	HS^−^_(aq)_	H_2_S_(aq)_	H_2_S (bar)
b	−74 ± 5	100 ± 4	123 ± 12
m	12.5 ± 0.7	−12.6 ± 0.5	−15.2 ± 2

**Table 4.** Change in energy for reactions associated with the chemistry of CaS nanoclusters in acidic media. Energy differences (ΔE) are reported in hartrees. The values in parenthesis represent the energy differences in kJ/mol.

Reaction	ΔE Hartrees (kJ/mol)
$\text{CaS}\to {\text{Ca}}^{2+}+{\text{S}}^{-2}$	0.067259 (6.48982091)
$\text{CaS}+{\text{H}}^{+}\to {\text{CaSH}}^{+}$	−0.48504 (−46.80170258)
$\text{CaS}+{\text{H}}^{+}\to {\text{Ca}}^{2+}+{\text{HS}}^{--}$	−0.45671 (−44.0679479)
${\text{CaSH}}^{+}+{\text{H}}^{+}\to {{\text{CaSH}}_{2}}^{2+}$	−0.4356 (−42.03075453)
$\text{CaS}+2{\text{H}}^{+}\to {\text{CaSH}}_{2}^{2+}+{\text{H}}_{2}\text{S}$	−0.91861 (−88.63629294)
$\text{CaS}+2{\text{H}}^{+}\to {{\text{CaSH}}_{2}}^{2+}$	−0.92064 (−88.83245711)

## Data Availability

Data is contained within the article.
